# AI for food: accelerating and democratizing discovery and innovation

**DOI:** 10.1038/s41538-025-00441-8

**Published:** 2025-05-22

**Authors:** Ellen Kuhl

**Affiliations:** Department of Mechanical Engineering and Bioengineering, Stanford University, Stanford, CA USA

**Keywords:** Biophysics, Engineering, Materials science, Physics

## Abstract

By 2050, feeding nearly 10 billion people will require transformative changes to ensure nutritious, sustainable food for all. Our current food system is inefficient and unsustainable. Traditional attempts to transform the global food system are too slow to drive innovation at scale. Here we explore the potential of artificial intelligence to reshape the future of food. We review the state of the art in food development, discuss the data needed to define a new food product, and highlight seven challenges where AI can help us design nutritious, delicious, and sustainable foods for all. By leveraging AI to democratize food innovation, we can accelerate the transition to resilient global food systems that meet the urgent challenges of food security, climate change, and planetary health.

## By 2050, we need to feed nearly 10 billion people

Until the middle of the century, the global demand for food is expected to increase by about one fifth^1^. Eradicating global hunger will require transformative changes to ensure equitable access to nutritious food for all^2^. But our current food system is broken^3^. It is a major contributor to environmental degradation, climate change, and food insecurity^4^. The global food system heavily relies on animal agriculture^5^, a leading source of greenhouse gas emissions, deforestation, and water use–yet, it remains inefficient at meeting the world’s nutritional needs^6^. To encourage consumers toward sustainable alternatives, European supermarkets are increasingly committing to shifting sales from animal to plant-based foods^7^: The leading European supermarket chain Lidl recently announced that it will increase its plant-based sales by 20% within the next five years, while the Dutch retailers Albert Heijn and Jumbo are targeting an even more ambitious ratio of 60 to 40 between plant-based and animal products by 2030^8^. It is increasingly clear that we need efficient and effective solutions. And we need them now^9^. This urgency has triggered the question: *Can artificial intelligence catalyze a paradigm shift in the global food ecosystem?*

## The potential of artificial intelligence is one of the most controversial topics of the 21st century

By all standards. In all aspects of modern life. And food science is no exception. But *Artificial Intelligence for Food?* Unarguably, the power of AI in food science cannot be overstated. It can be misunderstood, misused, or misinterpreted. But with the right understanding, use, and interpretation, it is impossible to ignore the massive impact AI will have on food science, discovery, and innovation^10–26^. The objective of this article is to examine the strengths, limitations, and future potential of AI for food.

## AI is critical to accelerate the revolution of our food system

Conventional methods are limited in their capacity to process and analyze massive amounts of data, making them too slow to drive innovation at scale^27^. To no surprise, we can observe an increasing use of AI in automating and optimizing food production systems^10^, improving sustainability and efficiency of land and water use^28^, and predicting and reducing food loss and waste^21^. On a personal level, AI can make dietary recommendations and guide personalized nutrition^25^; on a global level, AI can drive structural and systemic changes^18^. In the context of food innovation, the focus of this article, AI can enable the discovery of novel protein sources, optimize formulations for taste and texture^14^, improve production processes^19^, predict consumer preferences^22^, and create innovative products that mimic the nutritional profile, taste, flavor, or texture of animal-based foods^29^. At the same time, it is clear that today’s AI systems lack the ability to fully grasp the nuanced social, ethical, and sensory dimensions of food that are deeply rooted in human culture^30^. AI cannot entirely replace the human expertise, cultural understanding, and transformative creativity, needed to revolutionize our food system^31^. But there is hope that, through a synergistic partnership with AI, we can build healthier and more sustainable food futures, faster, cheaper, and more efficiently, with a speed and precision that is out of reach for traditional trial-and-error approaches today.

Before discussing what this perspectives article is about–the role of AI in shaping the future of food–let’s be very clear what it is *not* about: using AI as a black box solution without validating and interpreting its results; exploiting AI to overcome a shortage of data without understanding the quality, diversity, or representativeness of the data; and reducing AI to traditional tasks without exploring the true potential of AI for creative and generative solutions. Instead, the objective of this perspective article is to demonstrate that AI is a lot more than just a statistical tool for regression or classification:

*AI for food is a powerful technology to improve mechanistic understanding, inspire creative thinking, and democratize discovery and innovation towards reimagining food systems that prioritize health, sustainability, and justice for a thriving global population*.

## State of the art

### Traditional approaches towards creating new foods are too slow

**Fig. 1:**
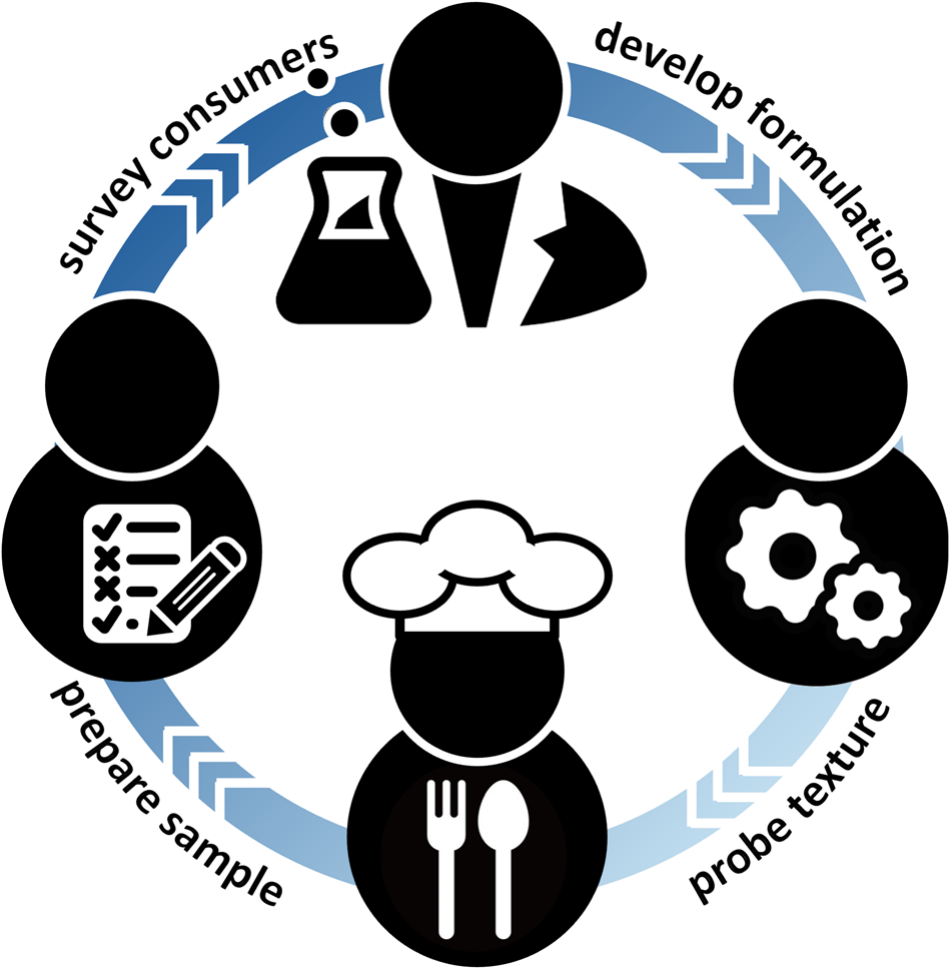
Traditional food development. Creating new foods is time-consuming, expensive, and inefficient. It involves iterative cycles of gradual improvement: food scientists develop a new production, engineers probe its texture and rheology, chefs prepare samples, and consumer researchers survey tasting panels for taste and flavor.

Creating innovative food products traditionally involves a complex process that combines food science, engineering, culinary art, and consumer research and heavily relies on iterative cycles of gradual improvement^32^. Figure 1 illustrates the food development cycle for the example of a new plant-based meat product^33^: The first step in the design process is to precisely *define the desired product* by identifying the target meat, e.g., chicken, pork, or beef; selecting the specific cut, e.g., burger, sausage, or steak; establishing key features, e.g., texture, flavor, appearance, or nutritional profile; and understanding consumer preferences, e.g., allergies, dietary restrictions, or environmental concerns. The second step is to *select the ingredients* by choosing protein sources, e.g., soy, wheat gluten, pea, or bean, to deliver the desired structure and nutritional profile; choosing fats and oils, e.g., coconut oil, canola oil, or shea butter, to mimic juiciness and mouthfeel; incorporating binders, e.g., methylcellulose or starch, and functional additives, e.g., carrageenan or lecitin, to enhance texture, binding, and stability; and adding flavors, e.g., yeast extracts or fermentation-derived compounds, to replicate umami or meaty flavors. The third step is to *develop the formulation* by optimizing the ratios of proteins, fats, binders, and additives to achieve the desired sensory attributes; addressing nutritional needs, e.g., high in protein, low in saturated fat, fortified with vitamins or minerals; and integrating flavor compounds to fine tune the taste, e.g., meaty, fatty, or smoky. The fourth step is to *engineer the texture* by choosing the processing method, e.g., extrusion, spinning, or 3D printing, to replicate the fibrous, layered structure of animal muscle; optimizing rheological properties, e.g., tensile, compression, or shear strength, to mimic the resistance to chewing; and designing a fat and moisture retention system to create the juiciness of the target product. The final step is to *optimize the product*, for example, by adding colorants, e.g., beet juice, annatto, or paprika, to improve product appearance; by performing customer surveys to satisfy texture and flavor preferences; and by improving product safety and shelf stability. By its very nature, this traditional approach involves dozens of cycles to develop formulations, probe texture, prepare samples, and survey consumers^22^. During these iterations, a change to *any* of the parameters in *any* of these steps can result in significant variations in the final product, which are often highly unpredictable. Obviously, this trial-and-error approach is time-consuming, expensive, and inefficient, especially when considering the urgency to transform our current food system. But fortunately, any of these steps provides an opportunity for AI: AI can drive ingredient selection^14^, formulation development^34^, texture engineering^35^, and product optimization^12^, and efficiently screen a massive multimodal parameter space to identify the most promising parameter combinations.

### How can AI accelerate the revolution of the food system?

Before we answer this question, it is important to understand that there are two different types of AI, non-generative and generative AI: *non-generative* AI analyses, improves, or infers data without creating new data, whereas *generative AI* creates new data that resembles existing data. Three traditional applications fall into the category of non-generative AI: *optimization*, probably the most widely used application of AI for food today, where the AI fine-tunes variables to achieve best possible outcome under certain constraints, for example, by optimizing ingredient combinations to maximize nutritional value and minimize environmental impact^19^; *discovery*, where the AI finds insights, patterns, and trends from data, for example, by identifying new protein sources from analyzing the chemical and mechanical properties of various plants to determine their suitability for mimicking the texture and taste of animal meat^11^; and *prediction*, where the AI forecasts outcomes or behaviors, for example, by predicting the taste of a combination of ingredients or the preference of consumers towards novel alternative protein products^36^. One very recent application falls into the category of generative AI^14^: *creation*, where the AI generates entirely new ideas, formulations, or textures^37^, for example, by creating entirely new formulations only on the basis of natural language prompts^38^.

### What exactly can AI optimize, discover, predict, or create?

To answer this question, let’s look at the example of replacing animal meat by an alternative protein product: The objective is to *discover* a new formulation for a product that either satisfies desired properties, e.g., nutrition, texture, or flavor, or mimics a specific target product, e.g., an existing, resource-intensive animal product that we seek to replace^34^. The new product also needs to satisfy certain constraints such as nutritional profiles^39^, texture^40^, and flavor^41^. We may also want to include or exclude water or specific ingredients, for example, to modulate texture or address food allergies. And we may want to include additional regional, seasonal, or environmental constraints^19^. From this input, the AI would *create* a set of new formulations as output, where each formulation consists of a list of ingredients with their respective fractions or weights. The AI could further *optimize* these formulations, for example, by constraining the number of ingredients, or reducing their environmental impact or cost. In addition, the AI could also *optimize* an associated set of process parameters^42^, for example, extrusion velocity and pressure, cutting, cooling, or heating, to achieve a desired texture and rheology. From the optimized formulation–encoded through the weighted ingredient list–the AI could *predict* properties, for example, the nutritional profile of the final product^43^.

### What are the current limitations of AI?

While nutritional profiles are relatively easy to predict from a list of weighted ingredients, it is a lot more challenging to predict rheology, texture, or flavor. This is not a general limitation of AI as a technology per se; rather, it is a temporary limitation that reflects the current lack of appropriate data or our inability to process big data at scale^44^. Using AI to generate new foods is still in its infancy, and data that correlate formulation to rheology, texture, and flavor are rare^15^. Labeled and structured data are often proprietary, as they require significant time, expertise, and resources to generate–especially in food science, where expert annotation adds substantial value^22^. Yet, only few approaches in the literature use *unsupervised learning*^45^ or *reinforcement learning*^46^, while most AI technologies for food today still heavily rely on *supervised learning* based on labeled data and human feedback^47^: Food scientists pilot production using the new formulation and process parameters, engineers probe its rheology and texture, chefs prepare it for sensory surveys, and consumers taste and annotate it for taste, flavor, texture, and overall customer satisfaction, similar to the graphic in Fig. 1. This laborious process might not immediately result in the most optimal product. Nonetheless, all the steps do naturally generate new training data that will provide useful information when creating future products. Ingredients, formulations, nutritional profiles, rheology, texture, flavor, and taste could constitute valuable data for a *foundation model*^48^, a large pre-trained multimodal model that understands relationships between these variables. Similar to many other applications of AI, we could envision a division of labor, where the process of building and pre-training the foundation model is performed by data science specialists while food science specialists would fine-tune the model to their specific needs.

## Data

### AI for food needs more data

By its very nature, AI, especially generative AI, is data hungry. This is no different when using AI for food. Fortunately, AI is inherently tailored to efficiently process and seamlessly combine data from many different sources^44^. To understand how AI integrates and processes multimodal data, let’s take a closer look at the types of data that characterize a food product inspired by a recent review that summarizes publicly accessible databases of mainstream food ingredients and recipes^14^. Let’s assume the AI encodes a food product by its *formulation, a list of ingredients with their respective weights*. We can assign each product vectors of ingredients, nutrition facts, taste, flavor, sensory texture, physical texture, and rheology. In addition, we could assign each ingredient a vector of its molecular structure and each product a vector of process parameters. We could also assign each ingredient a vector associated with its environmental impact and cost. Ingredients and nutrition facts are both listed on the food label; taste, flavor, and sensory texture are subjective measures of human perception; and physical texture and rheology are objective measures of physical quantities.

### Ingredients

**Fig. 2:**
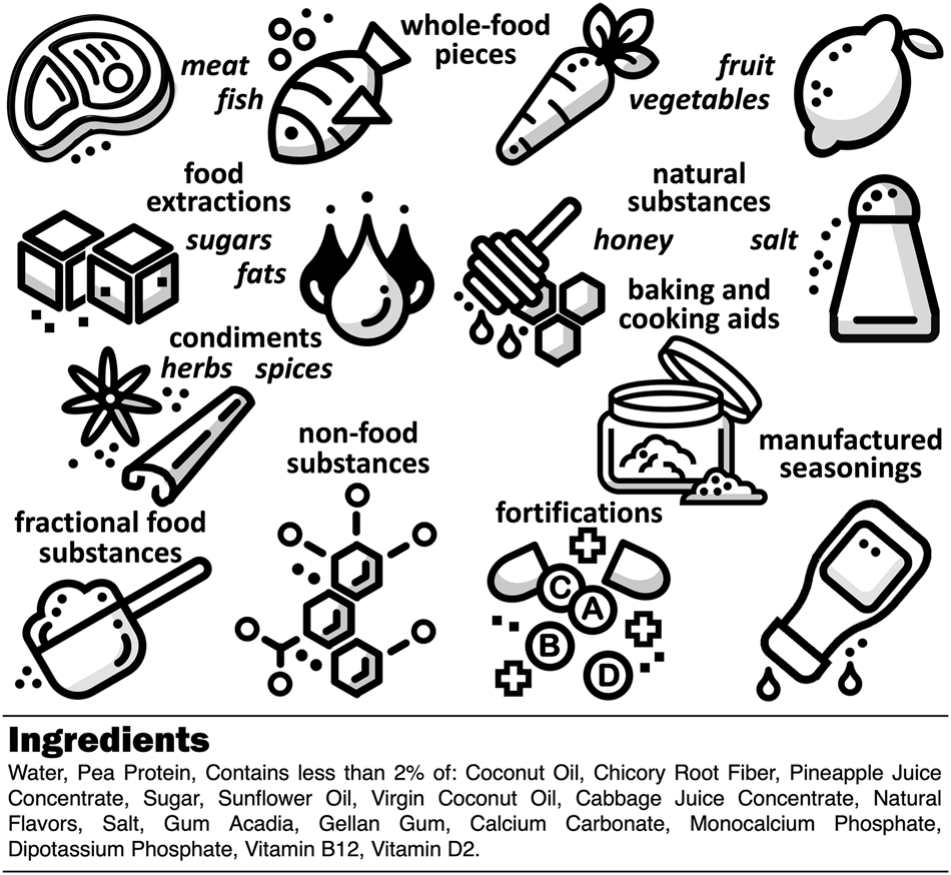
Ingredients are the functional building blocks of food. The ingredient list summarizes all ingredients in the product, including whole-food pieces, food extractions, natural substances, condiments, baking and cooking aids, fractional food substances, non-food substances, fortifications, and manufactured seasonings. The example provides the ingredient list for a plant-based milk product.

Ingredients are the *functional building blocks* of food^49^. The ingredient list on a food label summarizes all ingredients in the product, including primary food components, added ingredients, and other additives, in descending order by weight, meaning the most abundant ingredient appears first^50^. The ingredient list provides important information about what exactly is in the product, for example, to address food allergies or manage complex health conditions affected by particular ingredients^51^. We can broadly distinguish nine classes of ingredients as illustrated in Fig. 2: (i) *whole-food pieces*, e.g., from meat, poultry, fish, eggs, milk, fruit, vegetables, legumes; (ii) *food extractions*, e.g., fats, oils, sugar; (iii) *natural substances*, e.g., honey, maple syrup, salt; (iv) *condiments*, e.g., herbs and spices; (v) *baking and cooking aids*, e.g., raising agents, baking powder, vinegar, citric acid; (vi) *fractional food substances*, e.g., esterified oils, modified starches, isolated protein; (vii) *non-food substances*, e.g., food additives, flavoring substances, coloring additives, dough strengthener, enzymes, flavor enhancers, fat replacers, yest nutrients, firming agents, gasses, biologically active substances, protein substitutes; (viii) *fortifications*, e.g., added vitamins, minerals, fiber; and (ix) *manufactured seasonings*, e.g., sauces, marinades, dressings. A product is uniquely characterized by its formulation, the weighted list of ingredients. Importantly, the ingredient list alone is not sufficient to recreate the final product, since it only contains the ingredients, but not their fractions or weights. Yet, combined with the nutritional profile, it is possible to estimate the ingredient composition or volume fraction using optimization tools^43^.

### Nutrition

**Fig. 3:**
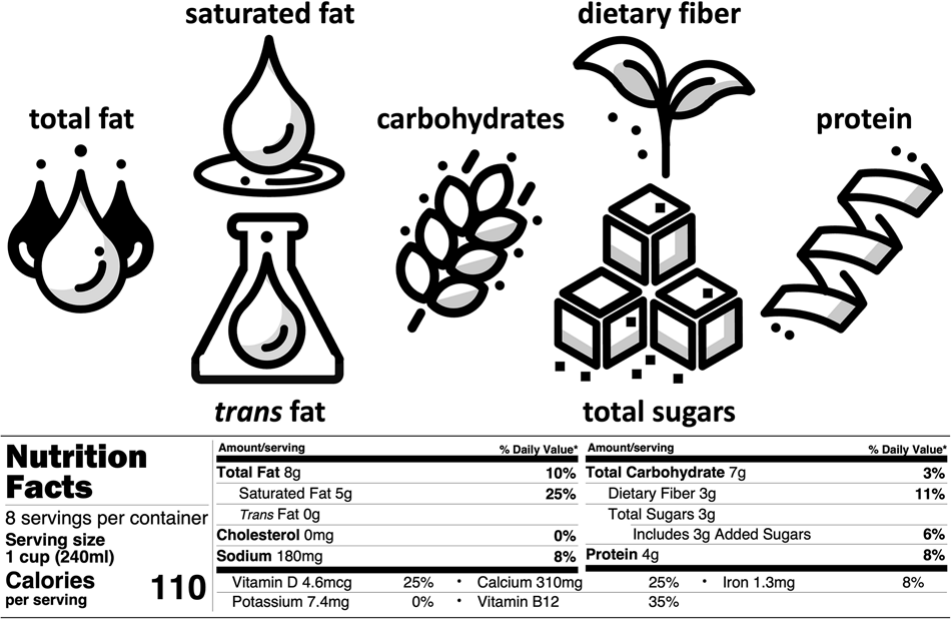
Nutrition is a functional property of food. The nutrition label contains information about macronutrients, including total fat, saturated and trans fat, carbohydrates, dietary fiber and sugars, and protein, and micronutrient,s including vitamins and minerals. The example provides the nutritional information for a plant-based milk product.

Nutrition is a *functional property* of food. In addition to the ingredient list, many countries require the nutritional facts to be part of the food label^52^. In the United States, nutrition information has been required on packaged foods since 1990, the first nutrition facts label appeared in 1994, and it has been updated to its current format^39^ illustrated in Fig. 3 in 2020. A nutrition facts label includes information about the *serving size*, total *calories per serving*, macronutrients including total *fat*, detailed into *saturated fat* and *trans fat*, cholesterol, sodium, total *carbohydrate*, detailed into *dietary fiber* and *total sugars* including *added sugars*, and *protein*, and micronutrients including *vitamins* and *minerals*. This information is typically provided both in units of weight and in percentage of daily value. Nutrition labels are commonly viewed as a low-cost tool to encourage healthy eating habits^53^.

### Taste

**Fig. 4:**
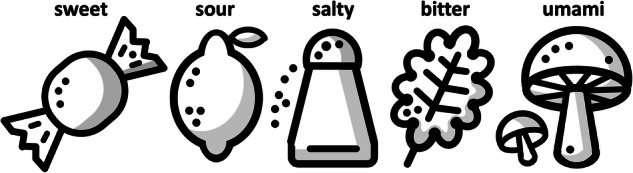
Taste is a sensory property of food. Humans can distinguish five basic tastes, sweet, sour, salty, bitter, and umami.

Taste is a *sensory property* of food that is triggered when molecules dissolve in saliva and interact with taste receptors of our tongue^54^. Humans can distinguish five basic tastes^55^ as illustrated in Fig. 4: (i) *sweet*, triggered by sugar or energy-rich compounds that we sense with the tip of our tongue; (ii) *sour*, triggered by acids such as citric acid in lemons that we sense at the sides of our tongue; (iii) *salty*, triggered by salts, like sodium chloride that we sense at the front of our tongue; (iv) *bitter*, triggered by toxins or alkaloids that we sense at the back of our tongue; and (v) *umami*, triggered by savory or meaty flavors, often from glutamates, that we sense in the middle of our tongue. While there is a general agreement on these five basic tastes, more recently, researchers have proposed additional taste categories such as fatty triggered by fatty acids, starchy triggered by complex carbohydrates, metallic triggered by metals or minerals, or spicy triggered by the sensation of pain. Recent studies suggest that artificial intelligence can classify the sweet, bitter, or umami taste of specific compounds based exclusively on their chemical structure^56^.

### Flavor

**Fig. 5:**
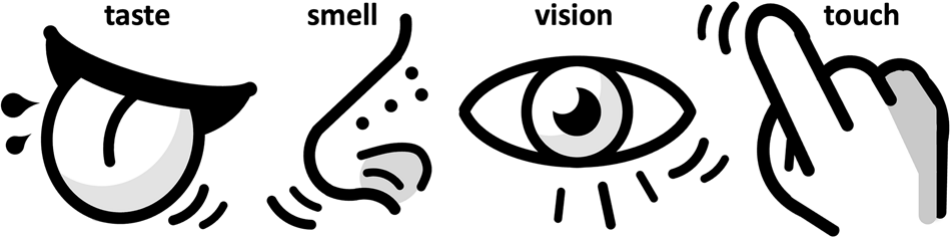
Flavor is a multi-sensory property of food. It arises from a combination of taste from the tongue, smell from the nose, visual appeal from the eyes, texture from mouthfeel and touch, and other sensory inputs collectively processed in the brain.

Flavor is a *multi-sensory property* of food^57^. Unlike taste, it integrates multiple senses^14^ as illustrated in Fig. 5. Flavor arises from a combination of taste from the tongue, smell from the nose, visual appeal from the eyes, texture from mouthfeel and touch, temperature, and other sensory inputs like spiciness perceived by pain receptors^58^. By collectively processing all these senses in the brain, humans can distinguish *thousands of complex flavors*.

An example is the flavor of pumpkin spice latte that arises from the interplay of the bitter-sweet *taste* from espresso and sugar, the pumpkin spice *smell* from cinnamon, nutmeg, and clove, the creamy, smooth, luxurious *texture* of milk, the warm and comforting *temperature*, the subtle *spiciness* of ginger, and the warm and cozy orange *color*, making it a multi-sensory delight, with each sensory input reinforcing the theme of warmth, indulgence, and the essence of autumn. Flavor directly affects our dietary choices and consumption patterns. Modern food science modulates flavors to promote a nutritionally balanced diet that is also healthy for our planet^14^.

**Table:** 

**Success story: Using AI to correlate chemical composition and sensory perception**. *Ajinomatrix* is an advanced AI-powered platform that provides an easy-to-interpret global model for taste and smell^59^. It leverages machine learning and advanced sensory data from consumer surveys, tasting panels, and sensors such as e-noses or e-mouths, to digitize taste, flavor, and aroma towards bridging the gap between chemical composition and human sensory perception. It is used by the food and beverage industry, food scientists, and flavor and fragrance companies to accelerate the design of new products with desired taste and smell.	

### Sensory texture

Sensory texture is a *sensory property* of food that refers to the subjective perception of the physical properties of food experienced by human senses^60^. As such, it is a qualitative characterization that is heavily influenced by personal perception^61^. Sensory texture is commonly assessed through consumer surveys or tasting panels that score about a dozen key features, which are loosely associated with physical texture and rheology^40^: *soft* and *hard* associated with the physical stiffness, hardness, and storage modulus; *brittle* and *chewy* and *gummy* associated with the physical cohesiveness and chewiness; *viscous* associated with the physical viscosity and loss modulus; *springy* associated with the physical springiness, resilience and plasticity; *sticky* associated with the physical adhesiveness; *fibrous* associated with the physical anisotropy; *fatty* and *moist* associated with fat and water content; and *meaty* associated with the perception of meat.

**Table:** 

**Fig. 6:**
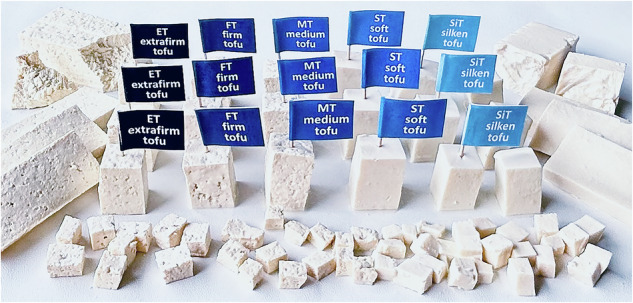
Correlating formulation and texture. The texture of tofu is directly correlated to its water content ranging from less than 76% to more than 90% to create five levels of texture, extrafirm, firm, medium, soft, and silken, from left to right.

**Example: Correlating formulation and texture**. One of the simplest examples of modulating texture through formulation is tofu, a versatile, protein-rich food product that is widely valued for its nutritional benefits. Tofu is produced by soaking and grinding soybeans to create soy milk, which is then coagulated using agents like magnesium chloride called nigari, calcium sulfate, or glucono delta-lactone. The resulting curds are pressed into solid blocks of varying firmness and texture^62^. The texture of tofu is directly correlated to its water content: the drier the firmer^63^. Food scientists distinguish five types of tofu illustrated in Fig. 6: *silken* with more than 90% water, about 4% protein, 2% fat, 1% carbs, and 44 kcal/100 g; *soft* with 87–90% water, about 6% protein, 3% fat, 2% carbs, and 63 kcal/100 g; *regular* with 82–86% water, about 8% protein, 4% fat, 2% carbs, and 79 kcal/100 g; *firm* with 76–81% water, about 11% protein, 5% fat, 3% carbs, and 102 kcal/100 g; and *extrafirm* with less than 76% water, 14% protein, 5% fat, 3% carbs, and 115 kcal/100 g.

### Physical texture

Physical texture is a *physicochemical property* of food that refers to a set of objective, quantifiable physical features that we can robustly and reproducibly measure using physical instruments^64^. The gold standard to characterize the physical texture of food is the classical texture profile analysis^65^, a double compression test from which we can extract six key features^66^: *stiffness* associated with the slope of stress-strain curve during first compression; *hardness* associated with the peak force during the first compression; *cohesiveness* associated with the material integrity during the second cycle compared to the first; *springiness* associated with the speed by which the material springs back to its original state after the second cycle compared to the first cycle; *resilience* associated with how well a sample recovers during the first unloading path compared to the first loading path; and *chewiness* associated with the resistance of a material during the chewing process. These six features of physical texture are proxies for our sensory experience of texture^67^. As such, they play a critical role as objective quantitative measures to predict consumer satisfaction and product quality.

### Rheology

Rheology defines the *physical properties* of food that characterize how food deforms and flows under physical forces that mimic the process of chewing^68^. These physical properties–elasticity, viscosity, and plasticity–influence processing behavior, physical texture, and, ultimately, our sensory perception^67^. The rheometer is the gold standard device to quantify the rheology of food through creep tests that measure material deformation at a fixed stress, relaxation tests that measure stress decay at a fixed deformation, shear rate sweeps that measure shear thinning or thickening, stress sweeps that measure yield stresses, and frequency sweeps that delineate whether a food behaves solid- or fluid-like^69^. The key rheological features include the *stiffness* *E* characterizing the reversible response, the *viscosity* *η* characterizing the dissipative response, the *yield stress* *Y* characterizing the transition from solid-like to fluid-like, the *storage modulus* ${G}^{{\prime} }$, characterizing the stored energy, the *loss modulus* *G″*, characterizing the dissipated energy, and the *phase angle* *δ*, distinguishing between elastic and viscous behavior^66^. Knowing the precise rheology is critical to understand how the product behaves during production, storage, and consumption^70^. By modulating any of these six rheological features, food scientists and food manufacturers can fine-tune the texture, consistency, and overall sensory appeal of the final product.

## Opportunities for AI

Artificial intelligence allows us to optimize many different aspects of food–fast, cheap, and efficiently–all at the same time. *Multivariable optimization* simultaneously optimizes a variety of variables such as nutritional content, taste or flavor, sensory or physical texture, rheology, environmental impact, or cost. We can assign each variable a different priority depending on region, culture, climate, or time of the year. AI can then help us evaluate the complex interactions between these variables and design new formulations that balance competing priorities, for example, taste vs. health, while satisfying consumer preferences and meeting production constraints.

But, realistically, how close are we to this goal? AI is already beginning to transform the speed, cost, and precision by which we develop innovative and sustainable foods. Yet, near term, it is unlikely that AI will fully replace all real-world trials, at least not in the foreseeable future. It is important to remain aware of the potential limitations of AI associated with a lack of transparency, a lack of computational power, and a lack of data. Most importantly, we should not become unrealistically optimistic and oversell the potential of AI for food. Instead, for now, we should leverage AI as a partner to systematically improve solutions, reduce the number of trials, and accelerate the timeline from seed to plate. Let’s take a look at eight challenges where AI is beginning to make a notable impact:

### Predicting and optimizing protein structures

Plant-based and cultured products require proteins with a specific structure to mimic the texture and taste of animal products. Similar to their use in drug development, generative AI models, such as generative adversarial networks^71^ or transformer models^72^, can predict and design novel protein structures that are optimized for specific needs, such as elasticity, chewiness, or binding capacity, or even bioactive compounds^73^ for human health^23^.

**Table:** 

**Success story: Using AI to discover bioactive compounds for human health**. *Brightseed*, a pioneer in exploring the potential of bioactives, used its proprietary AI *Forager* to discover two bioactive compounds, N-trans-caffeoyltyramine and N-trans-feruloyltyramine, as beneficial for gut health^74^. The phytonutrient development platform analyzed 700,000 compounds^14^, predicted the presence of N-trans-caffeoyltyramine and N-trans-feruloyltyramine in more than 80 plants, and identified hemp hulls as their richest natural source. This discovery led to the development of Brightseed®Bio Gut Fiber, an upcycled hemp hull product designed to support gut health and gut lining for healthy gut barrier function and prebiotic benefits^75^.	

### Discovering novel formulations

**Fig. 7:**
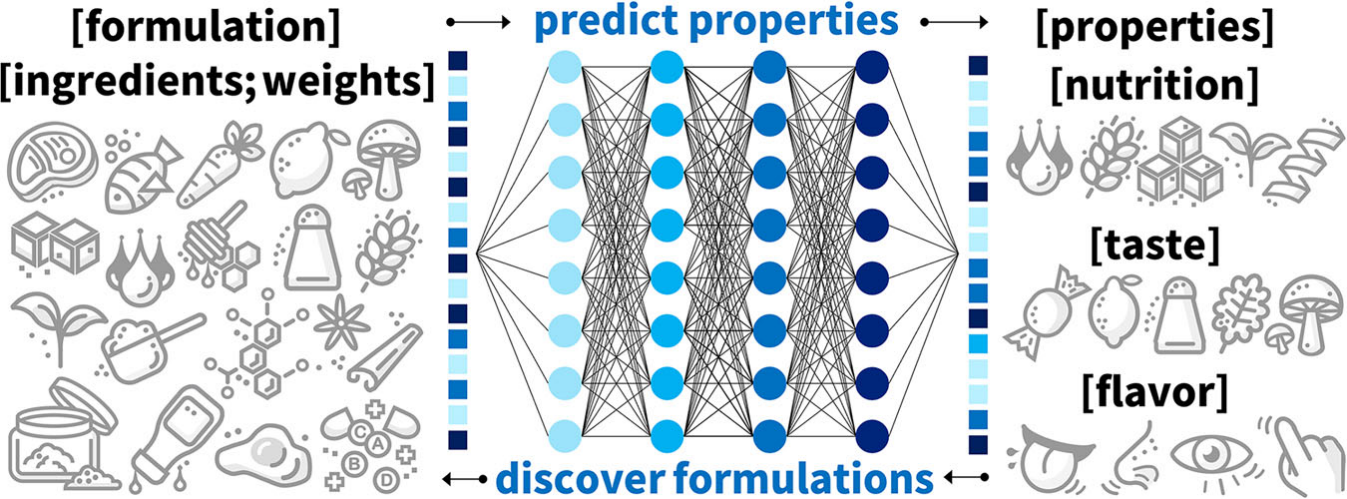
Artificial neural network to correlate formulations and properties. The network takes weighted ingredient vectors as input and creates property vectors as output. During training, the network learns the network weights and biases by minimizing a loss function, the error between the model and the data properties. Once trained, the network can predict properties for new formulations and discover formulations with desired properties.

Finding the right combination of ingredients to replicate the sensory experience of animal products is a time- and cost-intensive, challenging process^12,26^. AI can analyze massive datasets of food composition^16^, flavor profiles^14^, and consumer preferences^22^ to propose novel combinations of plant-based proteins, fats, and additives^24^. Figure 7 illustrates an artificial neural network that takes weighted ingredient vectors as input and creates property vectors as output. These property vectors can contain nutrition, taste, flavor, texture, and rheology, or process parameters, environmental impact, and cost. During training, the network minimizes a loss function, the error between the properties predicted by the model and the target properties of an existing product^34^. Once trained, we can use the artificial neural network for two complementary tasks: the forward problem of *predicting properties* for new formulations and the inverse problem of *discovering formulations* with desired properties^12^. For example, we could discover formulations for a new plant-based product that matches the properties of a resource-intensive animal product^76^.

**Table:** 

**Success story: Using AI to create new formulations for plant-based milk and chicken**. *NotCo*, a Chilean food-tech company, developed the AI platform *Giuseppe*^14^ that analyzed the molecular structure of animal-based products and discovered a combination of plant-based ingredients including pineapple juice concentrate, cabbage juice concentrate, and pea protein that can mimic the creamy texture and flavor of dairy milk^34^. This innovative approach led to the development of NotMilk®, an environmentally friendly and sustainable alternative to traditional dairy milk^17^. Figures 2 and 3 summarize the ingredients and nutrition of this plant-based product. Similarly, the platform discovered that the unorthodox combination of tomato and strawberry can mimic the texture of chicken leading to the development of NotChicken®. NotCo recently partnered with Kraft Heinz to transform their product portfolio towards creating delicious foods with simpler ingredients, faster development times, and reduced environmental impact.	

### Accelerating consumer testing

**Fig. 8:**
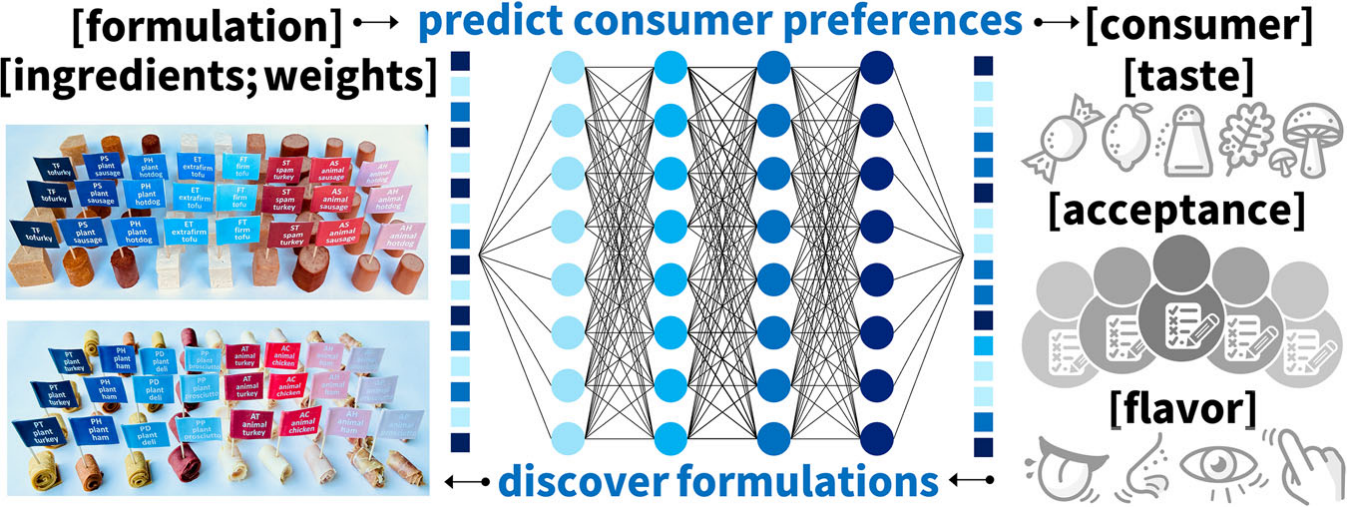
Artificial neural network to predict consumer preferences. The network takes weighted ingredient vectors as input and creates consumer preference vectors with taste, flavor, and acceptance as output. During training, the network learns the network weights and biases by minimizing a loss function, the error between the predicted model preferences and the consumer preferences. Once trained, the network can predict consumer preferences for new formulations and discover formulations for desired consumer preferences.

Gathering consumer feedback is expensive and time-consuming. Machine learning has emerged as an alternative technology to reduce the cost of sensory evaluation, enhance consumer satisfaction, and accelerate discovery and innovation^22^. Figure 8 illustrates an artificial neural network to predict consumer preferences. The network takes weighted ingredient vectors as input and creates consumer preference vectors of specific demographic groups as output. These consumer vectors could contain taste, flavor, appearance, texture, or acceptance rates. During training, the network learns the network weights and biases by minimizing a loss function, the error between the preferences predicted by the model and the preferences as scored by a consumer panel. Once trained, we can use the network for two complementary tasks: the forward problem of *predicting consumer preferences* of different demographic groups for new formulations and the inverse problem of *discovering formulations* for desired consumer preferences^12^. This reduces the need for extensive in-person trials and accelerates the time to market^26^.

### Replacing chemical additives and preservatives

The term *processed foods* is used for foods that have undergone relatively simple mechanical or chemical changes, for example, by cooking, drying, peeling, cutting, or cleaning, to improve nutrition, taste, or shelf life. In contrast, the term *ultra-processed foods* characterizes foods that have been industrialized and include a long ingredient list of chemical additives or preservatives that are not available for purchase on their own in supermarkets^51^. Especially plant-based foods are often ultra-processed to mimic the taste, flavor, and sensory signature of their animal counterparts^77^. Increasing evidence suggests that ultra-processed foods are unhealthy, both for the planet and for people^78^; yet, they contribute up to 60% of consumed calories in developed nations^79^. Here we can leverage AI in two ways: to predict the degree of processing and to substitute unhealthy ingredients by healthier alternatives^80^. For example, the machine learning classifier *FoodProX* takes nutritional features as input to *predict the degree of processing* on a scale from zero to one to provide consumers with information that could positively impact their dietary choices^20^. Alternatively, a recent review provides an overview of machine learning tools to *substitute ingredients* using rule-based techniques, vector embeddings, knowledge graph techniques, and other theoretical approaches^81^. Applications range from systematically replacing chemical additives, colorants, and preservatives in food formulations^82^ to replacing ultra-processed foods altogether by learning healthier food substitutes^83^ or discovering entirely new plant-based cooking recipes^45^.

**Table:** 

**Success story: Using AI to create a new formulation for plant-only ice cream**. The *Live Green Co* developed the AI platform *Charaka* that integrates ancestral plant knowledge with modern technology to revolutionize the food industry^84^. To date, it has categorized more than 15,000 plants. Using these data, the AI discovered a new formulation for an innovative plant-only ice cream made up of bananas, avocados, and sunflower seeds, a healthier and more sustainable alternative to traditional dairy-based ice creams. In contrast to *plant-based* ice creams, this new *plant-only* ice cream not just replaces the animal protein, but also other synthetic, processed, unsustainable, and unhealthy ingredients with more natural alternatives. Since most ingredients of the newly discovered formulation are not readily available in commercial form, the company uses precision fermentation^85^ to synthesize plant-based ingredients in a scalable way towards making food labels cleaner overall.	

### Predicting texture and mechanical properties

**Fig. 9:**
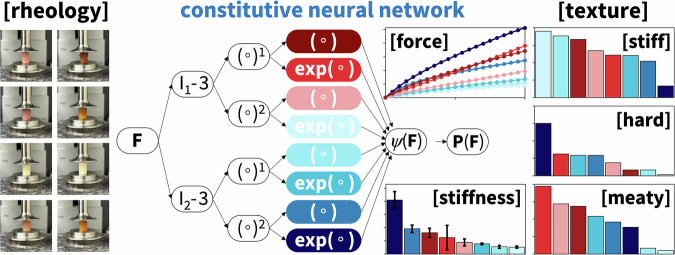
Constitutive neural network for automated model discovery. The constitutive neural network consists of a sparsely connected feed-forward network with activation functions that are reverse-engineered from the fundamental functional building blocks of popular material models. It maps physical deformations onto stresses. The network automatically discovers the best physics-based model and parameters to describe the data from texture profile analysis and rheological tests and reduces the cost, time, and bias associated with sensory surveys.

Traditional sensory and physical testing of food texture is time- and labor-intensive^66^. Figure 9 illustrates a constitutive neural network for automated model discovery of plant-based and animal meats^86^. The network takes deformation-stress pairs from texture profile analysis or rheological tests as input and discovers the best physics-based model and parameters to describe each individual meat^87^. In contrast to traditional sensory panels, automated model discovery is cost-efficient, easily reproducible, fast, and unbiased. Similar to its applications in materials science^88^, automated model discovery can discover and predict the rheological behavior of innovative and sustainable food products under tension, compression, and shear and provide insights into their microstructural architecture^89^. Predicting how these mechanical properties translate into our sensory experience would open new avenues towards refining formulations without extensive physical prototyping^35^.

### Enhancing flavor profiles

**Fig. 10:**
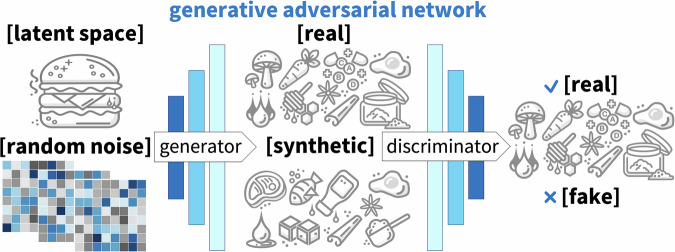
Generative adversarial network to create new formulations. The architecture consists of two sub-models, the generator and the discriminator. The generator creates synthetic data that mimics the real data, for example, real formulations of plant-based or animal meats. The discriminator distinguishes between real and fake data. During training, the generator and discriminator engage in a competition during which the generator strives to produce more realistic data and the discriminator aims to accurately classify whether the data are real or fake.

Plant-based alternatives often have undesirable off-flavors or lack the complex flavor profiles that we are familiar with from animal products^90^. Figure 10 illustrates a generative adversarial network that takes real formulations as input and generates novel formulations as output. The generative adversarial network consists of two sub-models, the generator that creates synthetic data that mimic the real data and the discriminator that distinguishes between real and fake^71^. Generative adversarial networks are known to produce valuable samples quickly, which we could then expose to the traditional cooking and sampling process^91^. We can leverage generative AI to analyze flavor-compound interactions and generate potential pathways to replicate umami, fatty, and smoky notes in plant-based or cultured meat products^14^.

**Table:** 

**Success story: Using AI to accelerate the transition to plant-based products**. *Knorr* by Unilever committed to transition 50% of its product portfolio to plant-based products by 2025. To achieve this goal, Knorr partnered with *Foodpairing*, a company that utilizes their proprietary AI to discovery new and complementary pairings of plant-based ingredients that work well together based on their flavor profiles^92^. The Knorr product development team uses these newly discovered formulations to create innovative plant-based products with desirable taste and texture. Leveraging Foodpairing’s AI allows Knorr to replace animal-based components without compromising taste and accelerate the transition to a more sustainable food system.	

### Generating new formulations from text prompts

**Fig. 11:**
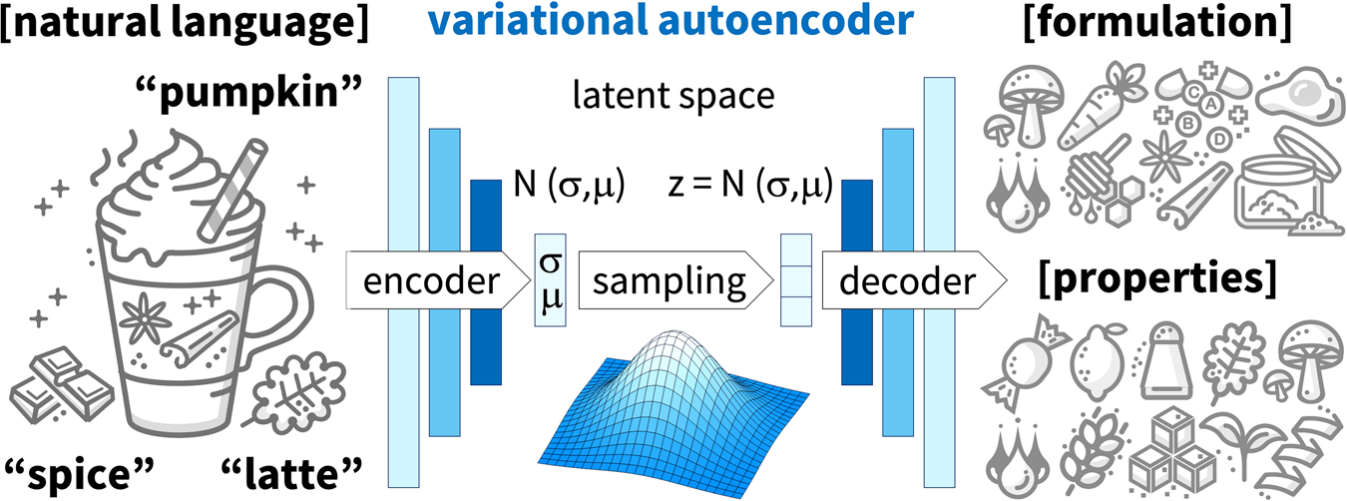
Variational autoencoder to correlate natural language and formulations. The autoencoder consists of two sub-models, the encoder and the decoder. The encoder translates inputs, for example, natural language prompts, into a consistent numerical representation. This representation makes up the latent space or design space. The decoder translates this numerical representation into outputs, for example, novel formulations with desired properties. During training, the model minimizes a loss function, the error between an existing formulation and the predicted novel formulation.

A critical unmet need in flavor development is the time-consuming, resource-intensive creation of new formulations, a process that critically relies on the experience of a small group of expert specialists^32^. Figure 11 illustrates a variational autoencoder that takes natural language prompts as input and generates novel formulations as output. The autoencoder consists of two sub-models, the encoder that translates the input into a consistent numerical representation, and the decoder that translates this numerical representation into outputs^93^. The numerical representation makes up the latent space or design space, which we can systematically explore to identify regions with desired properties^94^. Leveraging generative AI by generating new formulations from text prompts could significantly reduce the cost and time to market, address the industry’s constraints, and accelerate and democratize innovation^14^.

**Table:** 

**Success story: Using Generative AI to create new fragrance or flavor formulations**. *NotCo* recently presented it first *Generative Aroma Transformer* model that highlights the transformative potential of generative AI in product discovery with applications to flavor and fragrance formulations^38^. The model leverages a from-natural-language-to-chemical-composition framework by discovering novel molecular combinations for sophisticated aroma profiles, simply from text prompts such as cherry, candy, and vanilla. Trained on massive fragrance datasets, the transformer uses advanced graph neural networks to model molecular interactions and predict aroma outcomes. In blind tests, the artificially generated fragrances rival those crafted by human experts and address the shortage of certified perfumers. This innovation accelerates and democratizes flavor discovery and disrupts traditionally time- and resource-intensive industries.	

### Foundation models for food

Foundation models are large-scale, pre-trained models that learn generalizable representations across diverse data and enable rapid adaptation to specific downstream tasks^95^. To date, no such models exist in the context of food. A foundation model for food could integrate multimodal data– *structured* from ingredient lists, nutritional profiles, sensory and mechanical properties, and *unstructured* from images, cooking videos, and consumer reviews –into a unified architecture, often a transformer-based encoder^72^ with modality-specific embedding layers^96^. We would embed each food item into a high-dimensional latent space and train the base model on large amounts of data using self-supervised tasks, for example, masked feature prediction for recipes or ingredients, next-step prediction for cooking sequences, or contrastive learning for linking formulations and mechanical properties. For example, the recent foundation model *ChefFusion* that translates recipes into food images and vice versa was trained on more than 1 million recipes and 900 thousand images^48^. Once pre-trained, foundation models for food can be useful for numerous downstream applications, including inverse design^12^, optimization^14^, substitution^80^, and personalization^25^. In the context of this perspective article, we could envision using the foundation model to design new food products that mimic the nutrition, texture, and taste of animal-based products using alternative protein sources. This implies that we need to fine-tune the base model using the domain-specific data from Fig. 1, such as detailed experimental data on formulation, texture, and taste^67^, or paired mechanical tests and consumer surveys^87,97^. Once fine-tuned, the model could be used to predict sensory experience from ingredient lists, guide formulations toward target textures, or benchmark novel products against animal-based counterparts.

## Conclusion

The objective of this perspective article was to demonstrate the potential of artificial intelligence to transform our current food system. We have shown that AI provides a cost- and time-effective, scalable, and innovative approach to create high-quality alternative food products. As such, AI not only has the potential to accelerate the removal of animals from the global food system; it could also enable the synthesis of any desired food item from a range of environmentally friendly ingredients, and revolutionize the types of foods we consume and the methods by which we produce them. Such advancements could significantly enhance food system resilience, boost food security, and reduce greenhouse gas emissions in the benefit of human health and the health of our planet. The success of the proposed approach will critically depend on our willingness to share our results open source, and leverage the power of AI to analyze complex data, design solutions, and create new opportunities to democratize food science, discovery, and innovation for sustainable food futures.

## Data Availability

No datasets were generated or analysed during the current study.
